# Washback Effect of University Entrance exams in Applied Mathematics to Social Sciences

**DOI:** 10.1371/journal.pone.0167544

**Published:** 2016-12-09

**Authors:** Luis J. Rodríguez-Muñiz, Patricia Díaz, Verónica Mier, Pedro Alonso

**Affiliations:** 1 Department of Statistics and O.R., and Math. Education, Universidad de Oviedo, Oviedo, Spain; 2 CES San Eutiquio, La Salle, Gijón, Spain; 3 Department of Mathematics, Universidad de Oviedo, Oviedo, Spain; IUMPA - Universitat Politecnica de Valencia, SPAIN

## Abstract

Curricular issues of subject Applied Mathematics to Social Sciences are studied in relation to university entrance exams performed in several Spanish regions between 2009–2014. By using quantitative and qualitative analyses, it has been studied how these exams align with curriculum and how they produce a washback on curriculum and teachers’ work. Additionally, one questionnaire about teachers’ practices has been performed, in order to find out how the exams are influencing teaching methodology development. Main results obtained show that evaluation is producing a bias on the official curriculum, substantially simplifying the specific orientation that should guide applied mathematics. Furthermore, teachers’ practices are influenced by the exams, and they usually approach their teaching methodology to the frequent types of exams. Also, slight differences among the teachers lead to distinguish two behavioral subgroups. Results can also be useful in an international context, because of the importance of standardized exit exams in OECD countries.

## Introduction

Entrance exams to university (from now on, Spanish acronym PAU will be used, from *Pruebas de Acceso a la Universidad*) mean the main way of access to higher education, involving percentages over 70% from the total new freshmen to Spanish university [1]. They are into effect, with slight changes, since 1974. In the last performed model, under the Organic Law of Education [2], Ministry fixes some minimum requirements in Baccalaureate curriculum [3] that is later completed at regional level. Subsequently, the current structure of the PAU [4] was applied for the first time in the academic year 2009/2010. In every Autonomous Community (i.e., region) a committee composed by teachers and University professors designs and implements the exams by choosing the most influential topics in the assessment, the orientation of the questions, the level of domain and other specific characteristics of the exams.

Several years after the implementation of the new model, an important number of assessment units have been released, so that, it is possible to assess which concrete variations they have suffered in the subject Applied Mathematics to Social Sciences 2 (from now on, AMSS2, in the second year of *Bachillerato*, i.e., last year of upper secondary education,), which is the degree of fitting of the exams to the official curriculum, and how it all affects teachers’ practices.

Curricular guidelines orientate AMSS2 curriculum towards the need of solving real problems from the field of Social Sciences, within a proper context, and of teaching and learning Mathematics considering its instrumental essence in Social Sciences. Therefore, this kind of problems and exercises should appear in AMSS2 PAU exams. Problem solving and decision making skills are in almost all international curriculum for upper secondary mathematics, especially when they are applied to Social Sciences. The problem of how to reflect this type of problems in exit exams, external assessment or university entrance exams is, therefore, an international trend, as in many OECD countries this type of exams is being implemented (for an international comparative, see [5]).

Two are the main goals in this paper. The first one is to analyse the type of exercises and problems that are proposed in AMSS2 PAU exams and their relationship with the official curricula, in other words, the alignment between exams and the orientations in the official curriculum (note that it is fixed mainly by the national government and slightly completed by regional government, whereas exams composition involve both regional government and public universities in every region). Moreover, it is our interest to determine at what extent the posed questions have a clear relationship with applied problems, as it is stated in the official curricula.

The second goal is to study of the duality between official and real curricula, by analysing teachers’ practice. By using an *ad hoc* questionnaire, it is verified if PAU exams produce a washback in teachers’ activity.

These targets are addressed with the following research questions:

- How does PAU exams reflect official curricula in AMSS2? Are there biases within the exams?
- Does PAU influence AMSS2 teachers’ practice? How does this affect the implementation of the real curricula?

## Theoretical framework

Literature describes the effects of high-stake testing programs, defined as “tests whose results are used to trigger actions or decisions such as passing or failing a grade, graduating or not, determining teacher or principal merit or assuming responsibility for a failing district by a state agency” ([6]; cited by [7]). Apple [8,9] was one of the first authors identifying how centralized curriculum and assessment deskills teachers. Runté [10] enumerates these deskilling processes, emphasizing that centralized curriculum limits the range of skills required in making curricular decisions and it implies a shift from student-centred to curriculum-centred instruction. Smith [7] classified the effects of high-stake into 6 different categories, from which the present paper is only focused on types 4 (reducing the time available for instruction) and 5 (narrowing curriculum and reducing teachers’ ability to adapt, create, or diverge).

Jones et al. [11] conducted a study with North Carolina teachers concluding that high-stakes testing increase the amount of time that teachers dedicate to practice tests in their lectures. Besides, they confirm that “material that involves higher-order thinking and problem solving often falls by the wayside” [11].

Some authors support benefits from this type of curriculum-based external exit evaluations. Bishop [12] demonstrates how this type of exams improves students’ achievement in international assessments. Häkkinen [13] shows how Finnish university system would enlarge the proportion of university students if admission system were based on entrance exams instead of different admission criteria. Ou [14] demonstrates how students barely passing maths exams are more likely to dropout at university in the USA. On the other hand, Jacob [15] showed that the impact is not positive in general, by using a quantitative model relating scores on exit exams and university dropout. More specifically, other studies explore the field of mathematics [16–18] and also study differences between the official and the real curriculum (in the sense of Perrenoud [19]).

Spanish literature also reflects comparative studies about PAU and curriculum-based exit external evaluations in different countries or university entrance exams [20], some analyses about factors influencing the performance in Spanish exams and their predictive capacity [21–23]. Several studies have pointed out the influence of PAU mathematics exams on the definition of the real curriculum, methodologies, and, barely, teachers’ and students’ attitudes [24–27]. No references are found in literature considering curricular particularities of subject AMSS2. Some recent research have analysed probability and statistical inference problems in PAU exams in Andalusia [28,29], by using the onto-semiotic approach.

Regarding teaching practices, we follow the Mathematical Knowledge for Teaching framework (MKT) for characterizing teachers’ knowledge, developed in the group leaded by Ball [30]. According to this model, teachers’ practices belong to the Pedagogical Content Knowledge (PCK) domain, and, particularly, when examining practices in relation with high-stakes exams, we are studying the Knowledge of Content and Curriculum (KCC) and Knowledge of Content and Teaching (KCT). On the other, several authors have pointed out the influence of beliefs on practices, but also the difficulty, even impossibility, of measuring beliefs [31] by themselves, that is: “beliefs are referred to as constructed in the same sense that knowledge is constructed” ([31], p.128).

Assuming this theoretical perspective, it is also necessary to pay attention to literature regarding teachers’ practices related to high-stake exams. Bishop [12] showed how Canadian teachers tend to develop more complex tasks in the classroom in order to prepare graduation exams. Many studies have been developed trying to predict students’ performance based on several teachers’ characteristics such as experience or qualification (see, for instance, [32–34]). Recently, in [35] it is analysed how the type of classroom task and the amount of homework predict the outcomes in the Russian equivalent to PAU exam (USE).

In this research we are centred on the washback effect instead of focussing on students’ performance. The term washback has been coined in Educational Sciences to denote the influence of testing on teaching and learning ([36], p.259). It has been coined and especially used in language learning and assessing, but it can also be used in the mathematical context of the present work, since it also affects “curriculum materials, teaching methods, feelings and attitudes, learning” ([37], p.7). The concept itself can be used with different scopes (see, for instance, [38]), but in this paper it will be considered the effect on curriculum and teachers’ practices. When studying the effect of German Abitur exams, similar to PAU, [39] determine that: “It can thus be expected that, in the final years of schooling in particular, teachers align the standards of their in-class assessments with those of the upcoming central examinations.”

Therefore, our theoretical framework is built by integration of these previous approaches, and it is focused on the curricular analysis of AMSS2 related to the PAU exams, and on analysing the washback from PAU on teaching and learning methodologies. In summary: “It is testing, not the official stated curriculum, that is increasingly determining what is taught, how it is taught, what is learned, and how it is learned” ([40], p.88).

Additionally from previous sources, in the theoretical framework, from the methodological point of view, this work contributes with a novel approach to curricular analysis, paying attention to contents and their frequency appearing in PAU exams, and using a categorization inspired by conceptual focuses, developed in [41]. Since, for the present work, a broader classification than that in [41] is needed, the notion of curricular unit has been developed, and it will be deeply explained in the methodological section.

## Methodology

Considering that two different studies are developed, it is necessary to deal with two different samples.

### Sample I: PAU exams

Data from PAU exams have been collected in Andalusia, Asturias, the Basque Country and Madrid, from 2009–2010 to 2013–2014. Data were obtained from universities or regional education authorities web sites [42–45]. Asturias has been considered since it is authors’ original region, the Basque Country was interesting since it higher degree of autonomy, whereas Andalusia and Madrid have been considered since they have a great number of students in the exams. Therefore, the sample covers over 40% of the total number of students passing PAU exams every year, which illustrate its significance [46].

Each region has some degrees of freedom in the organization of PAU exams, what implies the number of exams is different among them. For instance, some regions as the Basque Country use the same exam for the two phases of PAU (both compulsory and optional ones) where as others as Asturias use different exams for both phases. Additionally, some regions release only the used exams, but other release also emergency exams (those used for extraordinary situations as blackouts, student accidents, etc.). Nevertheless, every exam has two different options, every option consisting in a set of exercises, so that the student has to choose one option and solve it completely, thus, for statistical purposes, we can consider each option as an exam. Hence, summing up, the number of analysed options, and the number of exercises included in options (usually 4 or 5, but it can also vary from one region to another and even from one year to another) are showed in Table 1. Actually, the whole population of exams in these 4 regions is studied, not a sample, since all the released exams have been analysed.

**Table 1:** Number of PAU exams and exercises analysed.

Region	Number of exams	Number of exercises
Andalusia	60	240
Asturias	40	160
Madrid	34	148
Baque Country	20	80
TOTAL	154	628

### Sample II: teachers

In order to analyse whether teachers’ practice in AMSS2 is conditioned by PAU exams, a sample of 51 Mathematics teachers in Secondary Schools has been used. The questionnaire was performed in two different periods: January-April 2013 and September-December 2014. Due to budget constraints, the sample was chosen with teachers from Asturias. They were selected by convenience sampling, by contacting those high schools in which future mathematics teachers were developing their internship. The 51 teachers belong to 21 different high schools (including public, private and state-funded). The University of Oviedo and the Regional Ministry of Education, within its program of Educational Research and Innovation, approved this research. This program implies that all results obtained can be used for research purposes, unless explicit disagreement from any involved agent and, hence, the procedure does not require the written consent, since all the research programs are publicized and approved by a research committee and another academic committee. Thus, teachers were informed about the use of the data and they gave oral consent to it.

### Instruments

Considering curricular contents, and assessment criteria defined by the Ministry and completed by the regional ministries, three observation tables have been designed in order to analyse content blocks in AMSS2 curriculum: Algebra, Calculus, and Probability & Statistics.

To register the information in an interpretable way, a new tool named Curricular Unit (CU, in the following) has been introduced. CU’s are defined as basic observation units for contents, procedures and assessment criteria in the curriculum. CU’s are constructed as an *ad hoc* simplification for our problem of the so-called conceptual focuses defined in [33]. CU’s have been designed by considering both so-called ‘contents’ and ‘assessment criteria’ paragraphs in the official curriculum. Therefore, CU’s arrange contents, procedures and assessment criteria into homogeneous curricular structures, allowing a coherent information retrieval and producing meaningful results about the frequency of appearance of each CU. Thus, CU’s not only are the tool to systematize the official curricula, but they also allow the observation of appearance frequency in each PAU exam, making it feasible to analyse the whole curriculum. This frequency observation is combined with a qualitative assessment of the type of problems and exercises posed in PAU exams, specifically, whether they propose solving contextualised problems and related with reality or not. Tables 2–4 show the defined CU’s for each block and its description.

**Table 2:** CU’s from Algebra block

A1: Matrices	Matrix language. Compile information using matrices.
A2: Matrices Operations	Basic operations. Solving easy linear equations using inverse matrix method
A3: Matrices Problems	Expressing natural language into matrix language.
A4: Range and determinant	Calculation and interpretation of matrix ‘range. Calculation and study of determinant´s properties. Relationship between range and determinant.
A5: System of equations	Solving matrix systems. Studying the number of solutions. Compatibility. Gauss’ and Cramer’s methods.
A6: Inequations	Linear inequations. Linear inequation systems. Graphic interpretation.
A7: Linear programming	Two-dimensional problems. Expressing natural language into linear programming problems. Feasible and optimal solutions.
A8: Applications of linear programming	Applications in solving social, economic and demographic problems.

**Table 3:** CU’s from Calculus block.

C1: Limits	Definition. Relationship between limit and tendency.Graphical interpretation of limits and asymptotic tendency with different kind of functions.
C2: Calculating limits	Rational, irrational, exponential and logarithmic functions.
C3: Real phenomena	Using functions to analyse and interpret real situations in Social Sciences.
C4: Continuity	Definition. Types of continuity. Continuity of polynomial, rational, exponential, logarithmic and piece-wise functions.
C5: Derivative	Definition. Interpretation. Calculus of the tangent line.
C6: Calculating derivative	Derivatives of polynomial, rational, exponential and logarithmic functions (maximum of two combinations). Using derivatives to study function´s local properties.
C7: Optimization	Using derivatives in optimization problems related to Social Sciences and economy.
C8: Studying functions	Studying local and global properties of polynomial and rational functions. Critical analysis of the information.
C9: Calculating integrals	Primitive function. Immediate integrals. Integration methods: integration by parts and by substitution.
C10: Definite integrals	Relationship between definite integral and primitive integral. Calculating easy surfaces and area under a curve by using integrals. Barrow´s rule.

**Table 4:** CU’s from Probability & Statistics block.

S1: Probability	Probability of simple and compound events. Laplace´s rule.
S2: Independent events.	Definitions and rules. Probability of dependent and independent events.
S3: Conditional probability	Definitions and contingency table. Probability of conditional events.
S4: Bayes and Law of total probability	Law of total probability, Bayes´ rule and tree diagram. Posterior probability.
S5: Making decisions with probabilities	Making decisions in Social Sciences problems involving probabilities
S6: Central limit theorem (CLT)	Central limit theorem (CLT). The normal approximation to binomial distribution Law of large numbers. Identifying the normal distribution. Determining the kind of distribution.
S7: Sampling	Choosing the best sample. Studying the representativeness of the sample.
S8: Confidence intervals	Probability distributions: mean and proportion. Confidence intervals for mean, proportion and difference between means.
S9: Hypothesis testing	Hypothesis testing for mean, proportion and difference of means. Studying significant differences between means or proportions of two populations.
S10: Inferring conclusions	Making decisions and obtaining conclusions about different situations.
S11: Distributions	Binomial and Normal distributions, probabilities associated to them.

This classification of the curriculum into CU’s is, from authors’ point of view, an efficient tool that allows analysing wide curricula, as it is in the case of AMSS2, and a huge number of exams, as presented here.

With respect to the second goal of this paper, to assess teachers’ practices one questionnaire has been designed, on the basis of the theoretical framework described above, and following Pajares’ statement: “beliefs cannot be directly observed or measured but must be inferred from what people say, intend, and do–fundamental prerequisites that educational researchers have seldom followed” ([47], p. 207). Therefore, questions ask about teachers’ practices regarding the teaching methodologies and their relation to PAU exams, the existence of practising tests, the influence of PAU exams on the real curriculum, the type of exercises and problems developed in the classroom, etc. The questionnaire consists of three thematic groups (questions appear in a later section)

- Group 1: Likert-type questions about the influence of PAU exams in their working methodology, selection of topics and assessment methods according to the subject AMSS2. Answers are considered being 1 = Totally disagree, 2 = Disagree, 3 = Neither agree nor disagree, 4 = Agree and 5 = Totally agree.
- Group 2: Open-ended questions about contents, competencies and suggestions and ideas to improve current PAU exams.
- Group 3: Questions about personal and professional data.

### Procedure and analysis

Data from the CU’s have been collected from PAU exams. Each exercise (out of the total 628) has been assigned to one or several CU’s. The assignment procedure consists of identifying the CU of every exercise in the whole set of exams.

Regarding the questionnaire about teachers’ practice, it was distributed in paper format among mathematics teachers belonging to the Departments of Mathematics at High Schools and having experience in teaching AMSS2. The questionnaire was delivered by the students of the Master’s Degree in Teaching Training of the University of Oviedo, during their internship period at high schools.

The information collected from the questionnaire has been treated with statistic package R, applying the following analyses:

- Descriptive analyses of each one of the three parts of the questionnaire.
- Quantitative studies about the possible relationship between data from the teachers (group 3) and their answers to the rest of questions (groups 1 and 2). It has been made through different non-parametric test (depending on the characteristics of the analysed variables), as there is no normality in the data, as it will be explained in the correspondent section of results. Correlations among different answers have been also considered.

## Results

### Curricular units of AMSS2 in PAU exams

The following tables show the percentage of appearance of each CU in the exams, with respect to the total amount of exams in the considered region. It should be noted that within each exam several CU’s appear, therefore the different percentages could sum up more than 100.

Table 5 shows clear differences in frequencies of Algebra CU’s. For instance, CU A3 (Problems with matrices) appears no more than 10% in three regions but in Asturias it reaches 42.5%. CU A4 only appears in Madrid (20.6% of cases there), whereas in the rest of the exams to obtain the matrix rank is never explicitly posed, neither the meaning of the rank nor its relationship with the determinant.

**Table 5:** Algebra: Appearance percentage of CU’s.

	A1	A2	A3	A4	A5	A6	A7	A8
ANDALUSIA	48.3	45.0	6.7	0	30.0	43.3	33.3	18.3
ASTURIAS	57.5	32.5	42.5	0	72.5	47.5	77.5	77.5
MADRID	44.1	32.4	5.9	20.6	67.6	20.6	38.2	23.5
BASQUE COUNTRY	30.0	40.0	10.0	0	10.0	40.0	50.0	10.0

Despite national AMSS2 curriculum include the specific assessment criteria: ‘To transcribe problems expressed in common language into algebraic language’ ([3], p.45476), it has been checked that only in the case of Asturias an important number of problems about linear equation system are set out in real contexts (CU A3), whereas in Madrid, Andalusia and the Basque Country most of the exercises are expressed without any context.

This situation appears partially replicated in the case of Linear Programming (CU’s A7 and A8). In Asturias all exercises deal with solving social, economic and demographic problems, however, in the rest of regions exercises provide the inequations, asking for their representation and for calculating the maximum or minimum of a given function, but most of the cases, mathematical formulation is not contextualised within a real problem, it is neither used the proper terminology of linear programming (objective function, feasible region, optimum solution, etc.).

Calculus block has less homogeneity, being the most frequent CU’s C5, C6 and C8, as it can be seen in Table 6.

**Table 6:** Calculus: Appearance percentage of CU’s

	C1	C2	C3	C4	C5	C6	C7	C8	C9	C10
ANDALUSIA	18.3	28.3	30.0	31.7	83.3	90.0	31.7	46.7	0	0
ASTURIAS	27.5	32.5	40.0	22.5	30.0	55.0	45.0	85.0	50.0	50.0
MADRID	38.2	47.1	14.7	35.5	73.5	82.4	14.7	64.7	47.1	70.6
BASQUE COUNTRY	5.0	10.0	30.0	15.0	45.0	90.0	50.0	80.0	0	35.0

Analysing by regions, in Andalusia it was never included any exercise related to integral calculus (CU C9), but in all exams derivate calculus is present (CU’s C5 and C6). Moreover, only 17 out of the 60 exercises have a formulation related to real life situations (CU C2). The rest are expressed mathematically without any context, and it is required to study some characteristics of the function (continuity, derivability, trends, etc.). In almost half of the exercises (24 out 60), the function is defined as a piecewise one.

In Asturias, there is an alternation in every exam between an exercise dealing with differential calculus (CU’s C5 and C6) and other dealing with integral calculus (CU’s C9 and C10). The first one is formulated in real life context (C7), whereas the integral calculus exercise follows always the same structure: calculating the primitive function and calculating the area under the curve, through Barrow’s theorem. The functions are usually polynomial.

Regarding Madrid, 22 out of 34 exercises combine differential and integral calculus, but the function is never defined in a real context.

Finally, the Basque Country does not include exercises related to integral calculus (CU C9), however most of the analysed exams present problems about differential calculus (CU’s C5, C6 and C7) and the study of functions (CU C8). Social phenomenon analysis only appears in 8 out of the 20 analysed cases.

Probability & Statistics block is the most heterogeneous one, as it is shown in Table 7, being CU’s S3 (Conditional Probability) and S4 (Bayes) the most frequent units. Several CU have barely appeared in the exams (CU S6: Central Limit Theorem practical applications, never appeared). The reason could be related to the difficulty to deal with Central Limit Theorem, except when considering it for calculating confidence intervals and hypothesis testing in great samples.

**Table 7:** Probability & Statistics: Appearance percentage of CU’s

	S1	S2	S3	S4	S5	S6	S7	S8	S9	S10	S11
ANDALUSIA	40.0	21.7	71.7	45.0	8.3	0	56.7	46.7	38.3	41.7	0
ASTURIAS	5.0	0	60.0	50.0	0	0	0	0	75.0	75.0	0
MADRID	29.4	14.7	52.5	37.5	5.9	0	85.3	82.4	0	0	11.8
BASQUE COUNTRY	35.0	10.0	10.0	50.0	10.0	0	0	35.0	15.0	0	45.0

All exercises in Asturias and the Basque Country are related to real life problems that need to be translated into statistic or probabilistic language. There are frequent themes such as alcohol consumption, loan granting, and health and work safety. However, in Madrid and Andalusia these contextualized problems are combined with others out any type of context.

In Asturias most exercises are focused on CU’s S3, S4, S9 and S10. But, there is an important number of CU never appearing: S2, S5, S6, S7, S8 and S11.

In Madrid, exercises focused on CU’s S7, S8, and S3 are the most frequent, the rest of CU’s having frequencies under 40%. In few exercises students have to make decisions about different probabilities or probabilistic scenarios, despite decision-making is one of the basic competencies in AMSS2. On the other hand, hypothesis testing is never posed.

In Andalusia confidence intervals are much less frequent than in Madrid, but, on the other hand, they include exercises about hypothesis and sampling, sampling distribution of the mean or sample representativeness.

In the Basque Country tests problems related to normal distribution usually appear in exams (CU S11) and problems related to total probability law and Bayes’ Theorem (CU’s S1 and S4). It is also easy to find exercises about calculus of probabilities through product rule, without further reflection about the sense of the operation.

### Influence of PAU on AMSS2 teachers’ practices

A questionnaire conducted on mathematics teachers in Secondary Schools in Asturias was performed. In this section the most important obtained results are presented.

#### Teachers’ personal and professional data

The questionnaire included a list of questions about social and demographic issues, besides other professional questions. The obtained results are listed below (N = 51):

- Sex: 41.2% of the interviewee were man, 54.9% woman and the 3.9% resting did not specify the sex.
- Age Rank: only one interviewee is between 22 and 35 years old, 19 are between 35 and 50 years old, 29 are over 50 years old and the two remaining have not answered to this question. It clearly reflects that Asturian teachers are quite aged.
- PAU experience: only 4 people have participated in PAU exams, as graders (members of the committee), since 2010.
- 84.3% of the teachers have taught AMSS2. Besides this percentage, 94.1% of teachers in the sample have also taught lessons in the first year of Baccalaureate (AMSS1).
- Regarding the experience teaching AMSS2, 9 people have never give lessons in AMSS2, 19 people have less than 5 years of experience, 12 people have between 5 and 15 years and the 10 remaining people have more than 15 years of experience. One of the teachers has not answered to this question.

#### Data from Likert-type questions

As it was specified in the description of the questionnaire, a second block contained 17 questions related to teaching methodology and its relationship with PAU, considered in a Likert-type scale from 1 to 5. Some questions are posed in a direct-affirmative way and some other in a negative way to avoid mechanical answering. Results are shown in Table 8.

**Table 8:** Answers to Likert-type questions in the questionnaire for teachers (N = 51).

Question	Median	Mean	SD
1. I do not teach some of the syllabus contents because they usually do not appear in PAU.	1	2.02	1.34
2. I pay more attention to questions that are usually asked in PAU.	4	3.88	1.09
3. I do not check the annual guidelines established by PAU exams coordinators.	1	1.50	0.91
4. I give up questions that are not usually asked in PAU when I do not have time enough to teach the whole syllabus.	4	3.76	1.23
5. I use the same methodology in both Baccalaureate courses.	3	3.59	0.95
6. I prefer teaching all contents within the lectures, instead of using more skill-oriented teaching methods in the second year of Baccalaureate.	3	2.88	1.08
7. PAU exercises determine my methodology in the classroom.	4	3.28	1.16
8. I usually do not use active methodologies in the second course of Baccalaureate.	3	2.67	0.97
9. In order to prepare my lectures I do not carry out an analysis of PAU exercises from previous years.	1	1.63	0.83
10. I consider exercises that usually appear in PAU exams do not influence my students´ learning.	2	2.57	1.31
11. Exercises that I solve in my lectures are usually similar to real problems.	3	3.13	0.91
12. In my exams I use exercises similar to PAU exercises.	4	4.06	0.92
13. I do not make any simulacrum about PAU exams.	2	2.39	1.31
14. If PAU exams did not exist, my students’ learning would fit more properly to curriculum	3	2.86	1.06
15. PAU exams do not produce a significant lack of competence in the academic training of students.	4	3.86	1.05
16. I would propose a different kind of exercises in PAU exams.	3	3.12	0.91
17. From my experience, PAU results in AMSS2 are similar to students’ results in Baccalaureate.	4	3.76	1.05

#### Results from open questions

The interviewees have given only four answers in this part of the questionnaire:

- In PAU exams students are not assessed on information and communication technologies.
- Students have a narrow basis from compulsory Secondary Education (previous stage to Baccalaureate) in Probability & Statistics.
- PAU exercises are very repetitive, more open problems should be included, instead of solving repeatedly procedural exercises.
- There is a lack of time to develop the official curriculum due to its extension, and this fact affects methodology, avoiding the development of cooperative learning method or researching techniques.

#### Results from quantitative data analysis

In order to complete the analysis, it have been analysed the existence of statistical relationship among demographic and professional data of the teachers, their answers to the Likert-type questions from the first block and the open ended questions from the third block.

For every Likert-type question and its possible relationship with demographic and professional variables, Kruskal-Wallis non-parametric test was used, as it was previously checked that answers were not normally distributed. This test allows determining if the differences between the different age ranks or years of teaching experience influence the answers of the Likert-type questions.

Results from Kruskal-Wallis test show in all cases that there is no significant relationship between any of the answers to Likert-type questions and the age, or between any of them and the years of teaching in the considered subject. Table 9 shows respective p-values in its last three columns.

**Table 9:** Obtained p-values for tests on Likert-type answers (Wilcoxon test in the first column and Kruskal-Wallis test in the rest).

P	Sex	Age	Teaching 1° Baccalaureate	Teaching AMSS2
Question 1	0.94	0.17	0.68	0.20
Question 2	0.89	0.36	0.89	0.51
Question 3	0.21	0.80	0.44	1.00
Question 4	0.84	0.48	0.34	0.24
Question 5	0.92	0.58	0.50	0.69
Question 6	0.52	0.92	0.37	0.82
Question 7	0.50	0.04	0.41	0.28
Question 8	0.48	0.18	0.19	0.50
Question 9	0.85	0.47	1.00	0.27
Question 10	0.09	0.28	0.31	0.52
Question 11	1.00	0.16	0.90	0.98
Question 12	0.23	0.27	0.65	0.33
Question 13	0.54	0.46	0.68	0.18
Question 14	0.58	0.23	0.84	0.38
Question 15	0.11	0.53	0.89	0.85
Question 16	0.54	0.99	0.17	0.75
Question 17	0.57	0.96	0.86	0.41

Regarding sex variable and each of the Likert-type questions, non-parametric Wilcoxon test used, to detect differences between medians by sex, p-values are shown in the first column of Table 9. It was not found in any case significant relationship among the variables. Therefore, answers to Likert-type questions do not depend on the sex, age or professional experience of the teachers. There is only a p-value that is slightly under 0.05, which will be discussed later.

Relationship between open questions and demographic and professional variables was analysed through Fisher’s (in the case of sex) and Barnard’s tests (for the rest of variables). It was chosen these kinds of non-parametric tests because results did not have the minimum number of values needed to apply χ2 test, and regrouping them would lead to nonsense. Obtained p-values are shown in Table 10. Again, results underline the consistency of teachers’ answers, which do not depend on the sex, age or years of professional experience.

**Table 10:** Obtained p-values from tests on answers to open questions (Fisher test in the first column and Barnard test in the rest).

P	Sex	Age	Teaching 1° Baccalaureate	Teaching AMSS2
Open Answer 1	0.31	0.27	0.56	0.31
Open Answer 2	0.14	0.40	0.44	0.46
Open Answer 3	0.29	0,79	0.45	0.18
Open Answer 4	0.50	0.85	0.32	0.54
Open Answer 5	0.75	0.70	0.30	0.30
Open Answer 6	1.00	1.00	0.28	0.52

Additionally, it was made an analysis of the correlations between the answers to Likert-type questions, in order to detect behavioural common patterns. In Table 11, correlation coefficients are shown between the answers to every pair of Likert-type questions. As it can be observed, despite being a sample of relatively small size, it appears significant correlations (over 35%) of direct relation among questions Q1-Q14, Q2-Q4, Q2-Q7, Q2-Q12, Q4-Q7, Q5-Q6, Q5-Q10, Q5-Q13, Q7-Q8, Q7-Q12, Q12-Q15, and Q15-Q17. It also appears important inverse correlations (under –30%) between the questions Q2-Q13, Q3-Q17, Q4-Q10, Q4-Q13, Q7-Q10, Q9-Q17, Q9-Q15, and Q12-Q13. In the discussion section the interpretation of these values will be included with more details.

**Table 11:** Correlation coefficients among answers to Likert-type questions (in percentage).

Q2	Q3	Q4	Q5	Q6	Q7	Q8	Q9	Q10	Q11	Q12	Q13	Q14	Q15	Q16	Q17	
22	-5	30	8	26	29	-8	2	-6	-15	16	-10	35	1	-1	9	Q1
	-3	54	21	13	48	30	0	-31	-7	42	-33	1,8	26	-25	-6	Q2
		-7	20	-28	-3	15	0	22	-13	6	14	20	-14	21	-35	Q3
			-10	-14	36	17	0	-48	-19	27	-34	27	11	4	-4	Q4
				39	-2	22	0	35	-19	8	38	2	16	-11	-20	Q5
					22	30	-3	-9	-26	5	29	18	-12	-13	13	Q6
						42	0	-41	1	54	-25	21	15	-10	-3	Q7
							0	-11	-25	25	4	-11	8	-18	14	Q8
								26	-4	-51	31	-15	-33	-6	-42	Q9
									6	-17	18	-12	25	-1	16	Q10
										27	-15	-20	32	-20	13	Q11
											-43	8	38	-14	21	Q12
												-11	-14	11	-18	Q13
													-27	17	-29	Q14
														-16	35	Q15
															-7	Q16

Analysis of correlations together with answers to Likert-type questions lead to classify questions into several subgroups:

- Considering Q1 and Q14 it is concluded that near 60% of teachers deny leaving parts of the curriculum without teaching when they are not usually asked in PAU exams (Q1), but, on the other hand, around 50% of them acknowledge that, if PAU did not exist, their teaching would be closer to the curriculum (Q14).
- However, more than 70% of the teachers do not deny that in case of being force to renounce to some parts of the curriculum, they would supress the questions that appear less in PAU exams (Q4). Moreover, Q4 has a strong positive correlation with Q2 (‘I pay more attention to questions that are usually asked in PAU’) and Q7 (‘PAU exercises determine my methodology in the classroom’). Additionally, Q7 is positively correlated with Q2, Q8 (‘I usually do not use active methodologies in the second course of Baccalaureate’) and Q12 (‘In my exams I use exercises similar to PAU exercises’). This shows how teachers try to teach the entire curriculum, but in case of lack of time, PAU contents are the chosen ones, keeping a methodology and a kind of exercises similar to PAU exams. Therefore, PAU causes a washback on the official curriculum.
- The inverse correlation between Q2, Q4 and Q12 with Q13 gives consistency to the described model, as this question is posed in a negative way. Therefore, there is a strong relationship between PAU and the day-by-day work in the classroom, establishing a quasi-logical relationship [48] between its preparation and the activity in the classroom.
- Despite previous answers, Q10 offers a not so clear result: about 25% of the interviewees believe that PAU does not influence students’ learning. Moreover, this question is negatively correlated with Q4 and Q7. This fact is analysed as the reluctance of teachers to admit a clear influence of PAU through a direct asked question, whereas when it is asked indirectly (Q2, Q3, Q5, Q7, Q9, Q12 or Q13) it is admitted with greater clarity. These answers show an internal conflict between their practices and beliefs, underlying that teachers are sensible in the way they enact their beliefs [49,50]. Teachers are also considering that their students usually perform in AMSS2 exams similarly to Baccalaureate, which is reinforced by Q17, showing a deep teachers’ support to the validity of the PAU exams and its prediction capacity with respect to grades obtained by students in the Baccalaureate [23].
- Something similar occurs with Q15, despite previous answers related to PAU influence, more than two thirds of the interviewees affirm that PAU does not cause significant distortion in students’ training. Thus, teachers consider that contents than can be avoided are not important in students’ training, showing a clear ratification of the coherence of the test.
- Finally, there are five questions (Q6, Q8, Q11, Q14 and Q16) where answers ‘Nor agree or disagree’ prevail. These are questions about active teaching-learning methodologies, competencies acquisition or using real problems that do not seem to raise a clear opinion among teachers. In the case of Q14, it is observed a small prevalence of the answer ‘Disagree’, which seems to highlight what has been underlined in the previous point. Q16 shows that most of the teachers do not express their opinion about changing the current PAU exam or maintaining it.

**Fig 1:**
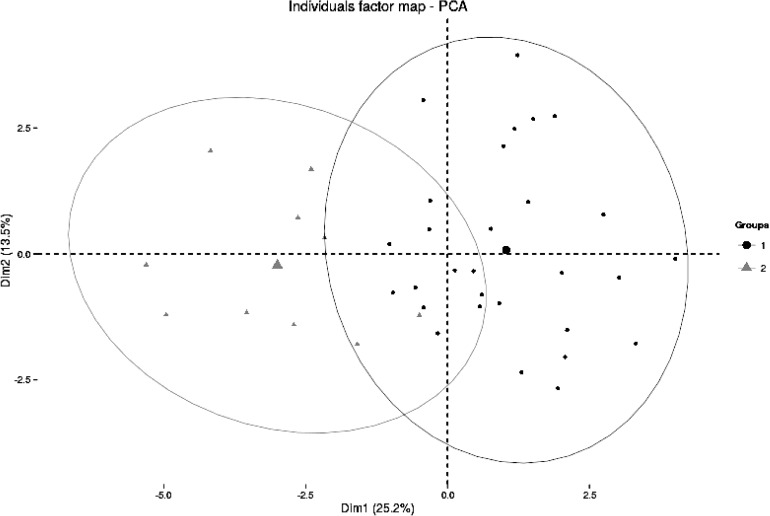
Cluster analysis. Clusters plotted over the two main dimensions in scores (biggest points represent group average)

Additionally, a cluster analysis has been performed over data from Likert-type questions. Results show how two main clusters can be distinguished. In Fig 1 the two main dimensions in scores are used as axes to plot the teachers’ scores showing both clusters. Nine individuals compose first cluster whereas the rest of the sample (42 members) belong to the second one. Main differences between the two clusters yield on scores in questions Q1, Q2, Q4, Q7, and Q12, with notably lesser scores for cluster 1, and questions Q9, Q10, and Q13, with higher scores for cluster 2 (maximum difference being 1.19 points in Q4). This fact shows that teachers in cluster 1 have a lower agreement degree with questions supporting the influence of PAU in their teaching methodology, whereas they have a higher agreement degree with questions denying that influence. Therefore, we could look at group 1 as teachers acknowledging less influence of PAU on their practice than those in group 2.

## Discussion

The first research hypothesis consisted on checking how PAU exams represent the official curriculum and on detecting possible biases on questions posed in PAU exams. Thanks to the study of all the exams released in the four considered regions it can be stated that although there are significant differences between the exams from the regions, there exist substantial parts of the official curriculum that are omitted or that appear underrepresented in all PAU exams. It has especial relevance that several CU’s have never appeared in the exams of any region, what implies a clear bias. On the other hand, there are some CU’s that have a constant presence in all analysed exams. Thus, it is demonstrated that PAU influences AMSS2 by producing a narrowed curriculum. This statement is consistent with conclusions in [27], but the present study is based on the study of all the released exams in four regions, being the first one doing this in the literature about this topic.

Another question that has been observed in the exercises analysis is the repetitive structure from one year to another within each region. This fact could imply positive effects, as it allows students organising and planning their learning, at this point, Wall [51] pointed out that “It should not be assumed that a ‘good’ test will automatically produce good effects in the classroom, or that a ‘bad’ test will necessarily produce negative ones” ([51], p.505-506). But this repetitive structure also has the negative side due to the high predictability of the exam, so students could limit their learning process only to the solving methodology of this kind of exercises with a scarce deepening: only learning to test. This fact opposes the understanding and the analysis of real life situations, as it is stated in the official curriculum. This result is consistent with [23] analysis for the particular case of the defined integral in PAU exams, but also with other research in the general field of high-stake testing. Moreover, this result endorses [10] into the deskilling processes of teachers, particularly math teachers with problem solving. But, on the other hand, PAU exams designing process is not including critical thinking as in Alberta exams ([10], p.173). Subsequently, it is necessary a proper alignment [52] between new curricular standards oriented to problem solving in real contexts and their assessment in central examinations, despite some tasks as critical thinking or decision making in mathematical frameworks have been underlined as very difficult to be evaluated in such type of exams [53]. This result is also consistent with [28], where a lack of contextualized probability problems was pointed out.

Additionally, assuming the lack of open problems and the pre-eminence of algorithmic exercises, there are few exercises including a context of close situations for the students, nor exercises describing phenomenon related to Social Sciences, despite AMSS2 official curriculum considers that a clear priority. Proposed exercises should stimulate reasoning (Jones et al. [11]), searching a solution and making a decision for the mathematical problem. Nevertheless, it should be underlined that PAU exams are limited in time to 1.5 hours, which could hinder to include open problems, which usually are considered without time limitation. This fact makes clear the crucial point about whether this type of assessment is the most adequate to such a curriculum [54]. This is also consistent with Kuhn [55] that underlines the difficulties of introducing context-based tasks in central examinations.

The management of the mathematical language used to model these situations and to express the solution of an exercise constitutes another important skill for the acquisition of a real mathematical competence. In this paper it is demonstrated that, mainly the Statistics & Probability block, some exercises needing translation from verbal to math language appear. This fact supposes an advanced in the direction of a greater didactic suitability of PAU exams, which was suggested in [26]. Nevertheless, research about difficulties and mistakes made by students in certain topics must be taken into account to design this curriculum-based assessment, for instance, in Statistics [29] points out the high difficulty of hypothesis testing for Baccalaureate students.

Assessment is a crucial point in the teaching-learning process, as it can condition learning processes and, moreover, it requires teachers’ adaptation to assess competencies. If extensive and general assessment procedures, as it is PAU, do not reflect this paradigm change from content to competence assessment, teachers’ role will be reduced.

The second research hypothesis was to check whether PAU influences AMSS2 teachers’ practices and to determine at what extent the real curriculum resulted from this behaviour. The research confirms that, despite there is not any explicit acknowledgment of it, PAU exams produce an influence on teachers’ practices in their day-by-day, and, what is more important regarding this paper, they confirm the washback on curriculum. This conclusion is derived from the analysis of the questionnaires.

From author’s point of view, results derived from answers to the questionnaire are noticeably different from a similar study in other region [27]. In both cases, teachers point out that their work is not conditioned by PAU, but in the questionnaires employed for the present paper it can be clearly remarked a greater utilitarian use of the teaching-learning processes in the second year of Baccalaureate towards the preparation of PAU exams. Besides, the present work contributes with the novelty of analysing a curriculum that has a defined orientation to the use in real life or Social Sciences frameworks of mathematics and, therefore, it is needed to pay more attention to the notion of mathematical competence, understanding mathematics as a tool to solve Social Science problems. Actually, this reinforces the results obtained in [35], establishing that practice test do not improve the performance, on the contrary, reflexive homework tasks increase it.

Besides, statistical analysis backups the homogeneity of answers among teachers, as there are no significant differences due to the age, sex or the years of professional experience in the subject. Therefore, perception respect to the importance of PAU are strongly consistent among teachers and, thus, results make clear PAU produces a washback in AMSS2 curriculum, not only on contents but also on methodology. Only few teachers answer with certain different responses, as it is demonstrated by the performed cluster analysis, so that, a small group can be distinguished from the rest by a lower degree of disagreement about washback of PAU. Authors are convinced this is a field that really needs an intervention, in order to reinforce teachers’ beliefs and about the importance of their practices, as [56] confirmed in the case of Finnish teachers, there is a strong relationship among mathematics teachers between their beliefs and their teaching practices. If a correct alignment is attained between new curriculum and exams, teachers’ practices will produce better effects on students’ self-regulated learning, as it was pointed out in [57].

Moreover, results open new ways for future research about teachers’ practices regarding the new final Baccalaureate exam that will substitute PAU in 2017 and, especially, the outcomes in this paper highlight some improvements that can be considered in designing the new exam. This is the moment to attain a proper alignment, being more faithful to innovations in the curriculum of AMSS2, especially those devoted to solve real (or likely real) problems, to use contextualized mathematics in the field of Social Sciences. It is also important to take into account the results to design a new much more balanced exam, not focused mainly on certain curricular units. Looking at the effect on teachers’ practices, results also point out the need of working into more varied type of exams, enhancing teachers’ flexibility to prepare and to manage the teaching/learning process, and releasing them from learning-to-test.

Finally, it is necessary to remark two main limitations in the present study. First, it would be recommended to widen the study of the exams to other regions (although the chosen sample considers four regions that suppose an important percentage of the scholar population in Spain). Second, the sample of teachers that have answered the questionnaire could also be widened to other regions, and be selected by a random sampling.

## Supporting Information

S1 File File containing data from teachers’ questionnaries. (XLS)
